# AI/ML-empowered approaches for predicting T Cell-mediated immunity and beyond

**DOI:** 10.3389/fimmu.2025.1651533

**Published:** 2025-08-29

**Authors:** Cheng-chi Chao, Yulun Chiu, Lucas Yeung, Cassian Yee, Chongming Jiang, Xiling Shen

**Affiliations:** ^1^ Terasaki Institute for Biomedical Innovation, Los Angeles, CA, United States; ^2^ ImmuX Consulting, San Jose, CA, United States; ^3^ Department of Melanoma Medical Oncology, Division of Cancer Medicine, The University of Texas MD Anderson Cancer Center, Houston, TX, United States; ^4^ Department of Immunology, The University of Texas MD Anderson Cancer Center, Houston, TX, United States; ^5^ Department of GI Medical Oncology, The University of Texas MD Anderson Cancer Center, Houston, TX, United States

**Keywords:** TCR-pMHC recognition, AI/ML-driven structure prediction, immunogenicity modeling, T-cell therapy design, protein-protein interactions

## Abstract

T cells play a dual role in various physiopathological states, capable of eliminating tumors and infected cells, while also playing a pathogenic role when activated by autoantigens, causing self-tissue damage. The regulation of T cell-peptide/major histocompatibility complex (TCR-pMHC) recognition is crucial for maintaining disease balance and treating cancer, infections, and autoimmune diseases. Despite efforts, predictive models of TCR-pMHC specificity are still in the early stages. Inspired by advances in protein structure prediction via deep neural networks, we evaluated AlphaFold 3 (AF3)-based AI computation as a method to predict TCR epitope specificity. We demonstrate that AlphaFold can model TCR-pMHC interactions, distinguishing valid epitopes from invalid ones with increasing accuracy. Immunogenic epitopes can be identified for vaccine development through in silico high-throughput processes. Additionally, higher-affinity and specific T cells can be designed to enhance therapy efficacy and safety. An accurate TCR-pMHC prediction model is expected to greatly benefit T-cell-mediated immunotherapy and aid drug design. Overall, precise prediction of T-cell immunogenicity holds significant therapeutic potential, allowing the identification of peptide epitopes linked to tumors, infections, and autoimmune diseases. Although there is much work to be done before these predictions achieve widespread practical use, we are optimistic that deep learning-based structural modeling is a promising pathway for the generalizable prediction of TCR-pMHC interactions.

## In-silico prediction of peptide-MHC binding and TCR recognition

There are many previous interests and attempts about predicting the antigen specificity in the T cell activity (1–19). Models for standalone prediction of peptide and MHC binding have existed for decades, such as NetMHC, NetMHCpan, MHCflurry, IEDB-AR, SYFPEITHI, TEPITOPE, MixMHCpred, DeepHLApan, PickPocket, MARIA, SMM, ARBO-MHC, HBond-MHC, MHCnuggets, PepCNN, BigMHC (20), and so on (21) (5, 21–30). Yet, for an assessment of antigen specificity and immunogenicity, the precise interaction of a given TCR to its corresponding pMHC complex should be further considered (31–33). Although tools, such as NetTCR (34), IMRex (35), ERGO (19), TEINet (36), AEPCAM (37), PanPep (18), pMTnet (38), TEIM-Res (39), PISTE (7), BERTrand (40), BigMHC (20), and HLAIImaster (41, 42), have been available, few models among them are able to accurately predict the recognition of pMHC complexes by T-cell receptors (TCRs) (31–33). The most precise method to dissect TCR-pMHC interactions involve experimentally generating X-ray crystallography structures, which is a time-consuming and technically demanding process. The primary hurdle in accurately predicting T-cell recognition of pMHC complexes lies in the inherent difficulties of protein structure prediction for TCRs and pMHC complexes. While numerous computational models employing various mechanisms have been developed to predict the structure of proteins like antibodies and TCRs, few of them have achieved satisfactory results (31–33). With the advent of the AI/ML era and models, many algorithms, such as AlphaFold 2 and AlphaFold 3 (43, 44), RoseTTAFold/RFdiffusion (45, 46), TrRosetta (47), HADDOCK (48), DeepFRI (49), CANDOCK (50), and Boltz-2 (51), have brought major improvements to predict protein structures and interactions. The accuracy of computational modeling has greatly improved. We have leveraged these advancements to explore AI/ML-driven computational protein structure prediction. Using our current model system, we can exploratively predict T-cell binding to pMHC complexes with significantly higher docking precision, enhancing reliability and biological relevance (14, 14, 31, 43–46, 52–58).

## AI/ML-powered computational design for predicting TCR-pMHC recognition

**Figure 1:**
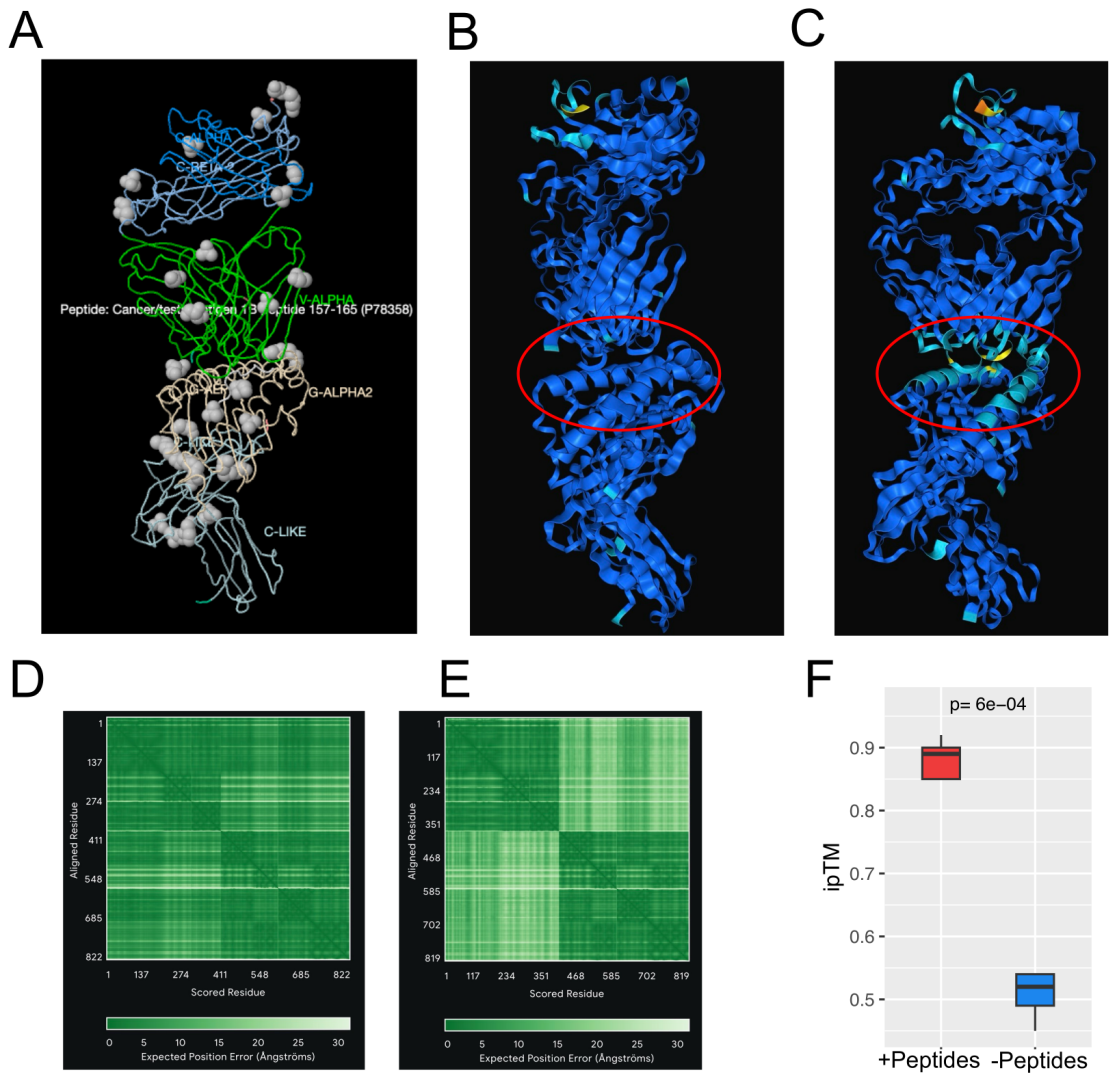
The x-ray crystallography structure and AF3-based prediction of T cell-pMHC interaction. **(A)** The x-ray crystallography structure of TCR binding to NY-ESO-1 derived peptide (SLLMWITQC)/HLA-A*02:01complexes (https://www.imgt.org/3Dstructure-DB/cgi/details.cgi?pdbcode=2PYE&Part=JMOL#jmolvisu) (60). **(B)** AF3 prediction of the interaction between specific TCR and NY-ESO-1 derived peptide (SLLMWITQC)/HLA-A*02:01 complexes. **(C)** AF3 prediction of NY-ESO-1-specific TCR binding to MHC molecules alone in the absence of its peptide epitope. **(D)** The predicted aligned error (PAE) of AlphaFold score, ipTM=0.92, from **(B)**. **(E)** PAE of AlphaFold score, ipTM=0.54, from **(C)**. **(F)** A total of 9 TCR-pMHC complexes (60–67) were included under both conditions: one group is the TCR-pMHC binding structure with peptides (+Peptides) and the other without peptides (-Peptides). All complexes involved the same class I MHC molecule (HLA-A*02:01) presenting 9–10mer peptides. The ipTM scores of the TCR-pMHC binding structure with peptides (+Peptides) are significantly higher than the TCR-pMHC binding structure without peptides(-Peptides), two-sided Wilcoxon tests, p-value =6e-04.

Computationally predicting TCR-pMHC interactions using AI/ML approaches offers a rapid, accurate, and scalable alternative to traditional experimental methods, thereby significantly facilitating antigen discovery and immune response modeling. AF3 is a publicly released model developed by DeepMind, trained extensively on more than 120 million protein sequences from the UniProt database and more than 2.2 million experimentally determined protein structures from the Protein Data Bank (PDB). We utilized the model as-is, without retraining or further fine-tuning. The default hyperparameters provided by AlphaFold 3 were employed, including three cycles of recycling, a multiple sequence alignment (MSA) depth of 256, and a template dropout rate of 15% (43, 44). A comparative analysis of AF3 with other structure prediction tools confirms that AF3 predictions outperform other tools in terms of structural accuracy and reliability (59). The results present a comparative analysis of TCR-pMHC recognition between the experimentally determined x-ray crystallography structure and AF3 computational predictions (**Figure 1**). The experimentally resolved crystal structure of the TCR-pMHC complex is shown in **Figure 1A**, serving as a reference for evaluating AF3 prediction accuracy. The crystal structure offers detailed insights into the spatial arrangement and interactions among the T-cell receptor (TCR) and peptide-MHC molecules. AF3’s prediction of TCR binding in the presence of peptide-MHC complex is shown in **Figure 1B**. This prediction closely mirrors the crystal structure in **Figure 1A**, demonstrating high accuracy in modeling the ternary complex. This highlights AF3’s ability to effectively predict TCR-pMHC interactions once the peptide is bound to the MHC groove. Conversely, AF3’s prediction of TCR binding to MHC in the absence of the same peptide (SLLMWITQC) is illustrated in **Figure 1C**. This prediction does not align well with the expected TCR binding conformation shown in **Figure 1B**. The reduced predictive performance from the AF3-based model highlights the importance of peptide presence for accurate binding predictions. This suggests that the conformation of the peptide-MHC complex is essential for accurate TCR interaction. The presence of the peptide resulted in a higher predicted interface template modeling (ipTM) score compared to its absence in AF3’s TCR-pMHC binding prediction (ipTM = 0.92 vs. 0.54, respectively) in the advanced protein structure prediction analysis, as shown in **Figures 1B, D** in contrast to **Figures 1C, E**. These high TM-score values confirm strong agreement between AF3 predictions and the crystal structure for the TCR-pMHC complex. The results demonstrated that the AF3-enabled approach reliably predicts TCR-pMHC interactions, supported by a high correlation with crystal structures and favorable TM-scores (**Figures 1B, D**). Instead, predictive accuracy decreases notably without peptides (**Figures 1C, E**). A comparative analysis in more TCR-pMHC binding structures assesses how peptide presence influences TCR-pMHC binding, as reflected in the ipTM values. In this analysis, the same set of 9 TCR-pMHC complexes (60–67) were included under both conditions: one group is the TCR-pMHC binding structure with peptides (+Peptides) and the other without peptides (-Peptides). The results demonstrate that the ipTM scores of TCR-pMHC binding structures with peptides are significantly higher than those without peptides (two-sided Wilcoxon tests, p-value =6e-04), as shown in **Figure 1F**. These findings highlight the significance of achieving accurate TCR-pMHC binding predictions with AF3.

## Future perspectives and challenges

AI-driven T-cell-pMHC modeling holds significant potential for drug discovery and clinical applications. However, a major hurdle in the existing models is their inability to accurately predict T-cell recognition of its cognate antigen (68–70). Developing in-silico tools to assess T-cell immunogenicity is crucial from both biological and therapeutic standpoints. Computational identification and screening of TCR specificity can greatly advance T-cell-related therapeutics, such as T cell therapy and vaccines, enhancing efficacy and safety (31–33, 40, 55, 68, 69, 71). A generalizable model of TCR-pMHC interactions can significantly accelerate the identification of dominant antigenic epitopes with high affinity for their respective MHC. Improving predictions of TCR binding to the pMHC complex can help fine-tune TCR affinity and address a key challenge in the field. Accurate predictions of the T-cell-pMHC complex structure can aid in designing agonistic or antagonistic peptide analogs to stimulate tumor-specific or tolerize (auto)antigen-specific T cells (34, 48, 72–75). For vaccine design, an AI-enabled model can assist in selecting suitable epitopes with strong immunogenicity, helping to accurately identify and validate those capable of activating T cells (76, 77). Moreover, accurate predictions of peptide-MHC interaction are expected to enable more effective assessment of the risk of anti-drug responses in patients, although current models often overestimate the results (34, 72–74). Ultimately, we hope that such a model will be able to predict T-cell functional activity in an exploratory manner, thereby contributing to the development of potential therapeutic strategies (**Table 1**).

**Table 1:** Exploring three key facets of therapeutic applications for accurate T cell–pMHC recognition prediction.

**I. Predict the binding of peptide epitopes to their respective MHC**
Screen and identify antigenic epitopes with potential strong immunogenicity for vaccine discovery.
Tailor the interaction of peptide-MHC interaction for designated therapeutic outcomes.
**II. Envision the interaction of antigen-specific T cells with their corresponding pMHC**
Predict TCR specificity from disease-associated T cells for target identification in T-cell therapy.
Improve therapeutic design by refining the T-cell affinity to enhance efficacy of therapeutics.
Reduce unintended cross-reactivity/off-target effects to minimize off-target effects for drug safety assessment.
**III. Predict the Magnitude and Nature of T Cell Responses**
Assess potential T cell responses to therapeutics, including both immunogenic activation and inhibitory effects.
Evaluate patient risk in developing anti-drug immune responses.
Assess the neoantigen quality of tumors and the associated resistance to immune checkpoint blockade therapy.

While the pharmaceutical and biotech industries have leveraged AI across various stages of drug discovery, primarily focusing on small molecules and antibody drugs, the application of AI/ML and digital biology to T-cell therapeutics remains relatively limited (68, 69, 76). As demonstrated by the results above, our advanced protein prediction modeling approach enables accurate prediction of T-cell-pMHC interactions, offering significant potential to enhance T-cell-mediated therapies. Despite advancements, AI-assisted protein design and protein-protein interactions (PPI) still face significant unresolved challenges (**Table 2**). Tools like AF3, Rosetta, and Boltz-2 have revolutionized protein engineering, yet several critical obstacles remain, as outlined below. One major challenge is the limited availability of high-quality data, especially for underrepresented antigens, rare HLA alleles, and paired TCR alpha and beta chains (42, 78–80). The lower the quality and quantity of training data available to AI systems, the less reliable their predictions of binding interactions become. Additionally, TCRs naturally exhibit a wide range of binding affinities and can be polyspecific, making it difficult to train models that accurately capture their complex interaction profiles (43, 44, 46, 52, 58, 81). A further complication arises from protein conformational dynamics. Proteins exist in multiple conformations; they open, close, twist, and bend. These conformational changes depend on factors such as temperature, pH, chemical environment, and interactions with other molecules (31, 33, 55). Moreover, TCR binding affinity alone is not sufficient to guarantee a functional immune response. A robust response requires a complex interplay of factors, including antigen processing and presentation, TCR binding,as well as T cell activation, differentiation and the diseased microenvironment (82–85). While AlphaFold can help discriminate between correct and incorrect binding partners, it doesn’t directly predict binding affinity in a quantitative way. Additional modeling approaches, such as Rosetta, are required to calculate binding energy changes, which can then be used to predict the effects of mutations on TCR affinity and correlate them with binding affinity. Ultimately, the development of a reliable and high-throughput screening system is essential for identifying effective therapeutic candidates in drug discovery. While our exploratory model system is an initial step, we aspire for it to drive future advancements in T-cell-based therapeutics.

**Table 2:** The major challenges in predicting TCR immunogenicity in silico.

**I. Limited availability of diverse, high-quality data for training**
Only a small fraction of potential TCR-ligand pairs available overall for model training.
Data on a diverse array of epitopes binding to TCRs needs to be generated. Most antigens reported to bind TCRs are viral, comprising the majority of TCR-antigen pairs.
Current datasets are dominated by antigens presented by common HLA alleles, with few under-represented HLA alleles included.
**II. Focusing solely on peptide-MHC binding without considering TCR interactions**
A strong peptide-MHC interaction may be necessary for T cell activation, but it is not sufficient to guarantee an immune response.
**III. Focusing solely on the β-chain CDR3 loops of TCR sequence information**
Both α and β chains contribute to antigen recognition and specificity. Incorporating both chains improves predictive performance, but chain pairing information is largely missing from current datasets.
**IV. Lack of INFO about the polyspecificity of individual TCRs**
TCRs can exhibit both specificity and promiscuity. Models that assume a given TCR recognizes only a single cognate epitope oversimplify the complexity of TCR-antigen interactions.
**V. Stronger TCR binding affinity alone does not necessarily translate to a stronger functional immune response**
Robust predictions of TCR specificity require a complex interplay of factors, including antigen processing and presentation, TCR binding, T cell activation, differentiation, and the diseased microenvironment.
**VI. The influence of thymic selection and self-peptide presentation is overlooked**
The naive immune repertoire formation is an under-explored area in TCR specificity prediction. The high affinity or immunogenicity of TCR predicted may not exist in periphery due to thymic deletion.
**VII. Limitations in Direct Quantification of Binding Affinity Prediction**
While AlphaFold can help discriminate between correct and incorrect binding partners, it doesn’t directly predict binding affinity in a quantitative way.
**VIII. The effect of hallucination of the AL/ML models will impact the precision of Binding Affinity Prediction**
The key factors, such as data quality, model regularization, fine-tuning, and better supervision, contribute to hallucination in the AL/ML models during binding affinity prediction.

## Data Availability

The original contributions presented in the study are included in the article/supplementary material. Further inquiries can be directed to the corresponding authors.
